# Supplementary material for: Historical and current spatiotemporal patterns of wild and vaccine-derived poliovirus spread
Source: Nat Microbiol. 2025 Nov 27;10(12):3148–61. doi: 10.1038/s41564-025-02174-6 (PMC12669034; doi:10.1038/s41564-025-02174-6)
Supplement: Supplementary file 1 — Supplementary Figs. 1–20 and Tables 1–5. [file 41564_2025_2174_MOESM1_ESM.pdf]

nature microbiology

Supplementary information https://doi.org/10.1038/s41564-025-02174-6

Historical and current spatiotemporal patterns of wild and vaccine-derived poliovirus spread

In the format provided by the authors and unedited

Supplementary Materials

Historical and current spatiotemporal patterns of wild and vaccine-derived poliovirus spread

Authors: Darlan da Silva Candido[1*], Simon Dellicour[2,3,4], Laura V Cooper[1], Carlos A Prete Jr[1,5], David Jorgensen[1], Christopher B Uzzell[1], Arend Voorman[6,7], Hil Lyons[6,7], Dimitra Klapsa[8], Manasi Majumdar[8], Kafayat Arowolo[8], Corey M Peak[6], Ananda S Bandyopadhyay[6], Javier Martin[8], Nicholas C Grassly[1], Isobel M Blake[1]

Affiliations:

[1]MRC Centre for Global Infectious Disease Analysis, School of Public Health, Imperial College London; London, UK.
[2]Spatial Epidemiology Lab (SpELL), Université Libre de Bruxelles; Bruxelles, Belgium.
[3]Department of Microbiology, Immunology and Transplantation, Rega Institute, Laboratory for Clinical and Epidemiological Virology, KU Leuven; Leuven, Belgium.
[4]Interuniversity Institute of Bioinformatics in Brussels, Université Libre de Bruxelles, Vrije Universiteit Brussel; Brussels, Belgium.
[5]Department of Parasitology, Institute of Biomedical Sciences, University of São Paulo; São Paulo, Brazil
[6] Gates Foundation; Seattle, USA.
[7]Institute for Disease Modeling, Gates Foundation; Seattle, USA.
[8]Division of Vaccines, Medicines and Healthcare products Regulatory Agency (MHRA), South Mimms, EN6 3QG, UK.

*Corresponding author. Email: ddasilva@ic.ac.uk

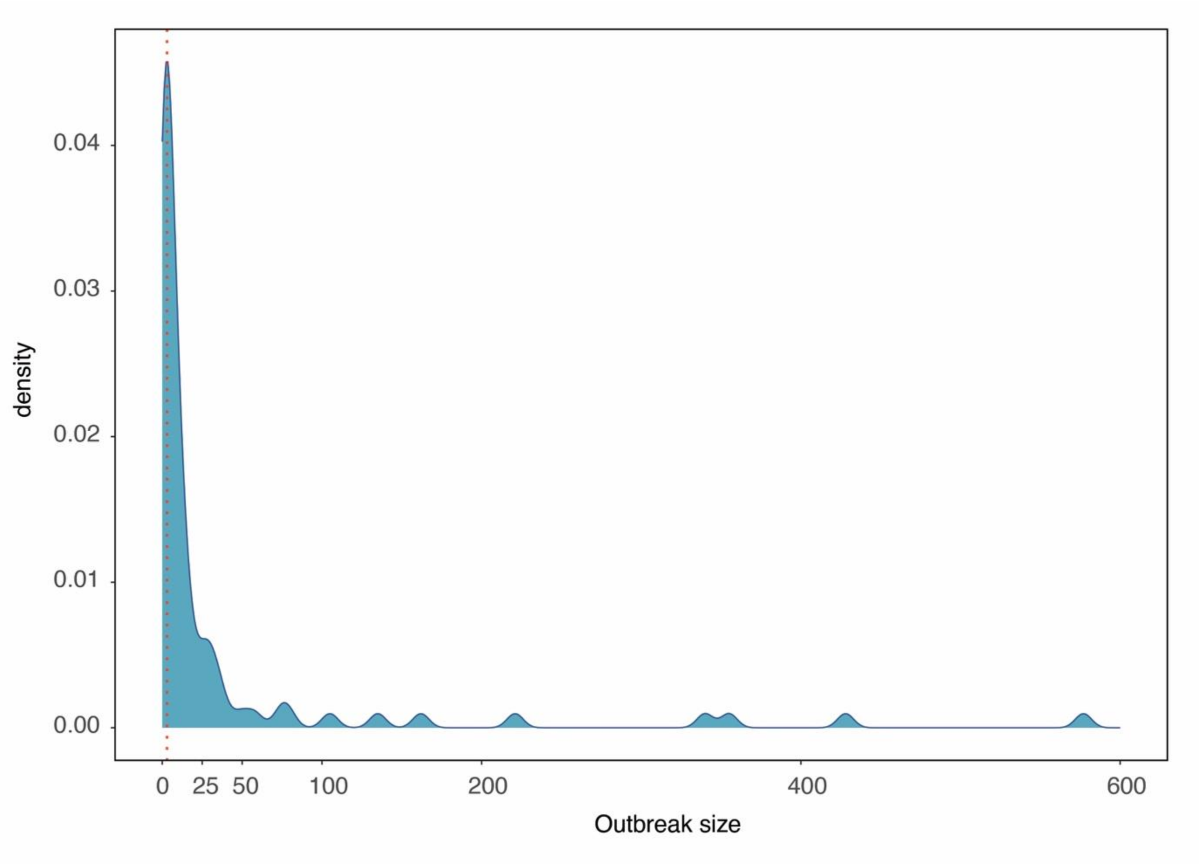

Fig. S1.
Distribution of cVDPV2 outbreak size based on the number of confirmed cVDPV2 AFP cases reported between May 2016 and September 2023. Dotted red line shows the median outbreak size, 4.5 cases.

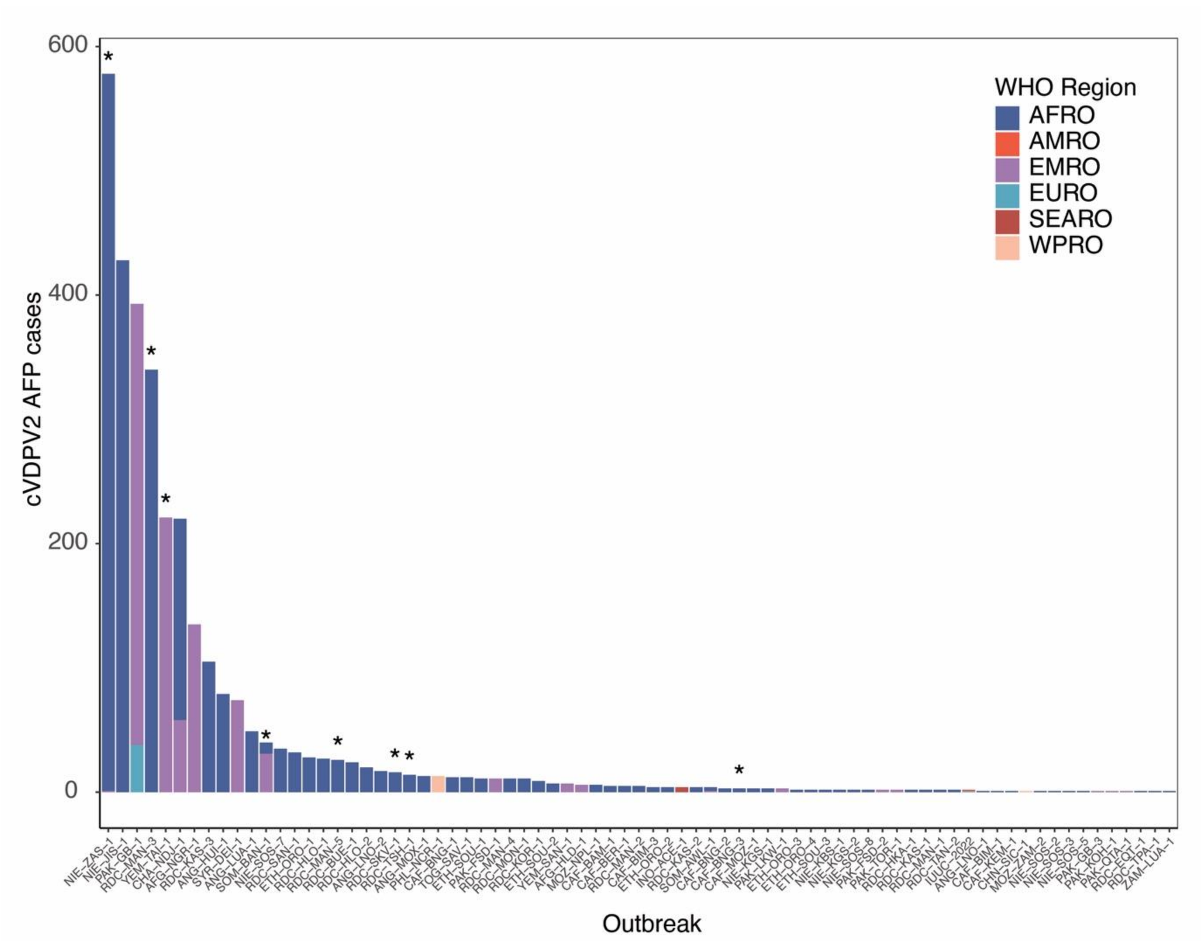

Fig. S2.
Distribution of number of confirmed cVDPV2 cases per outbreak reported between May 2016 and September 2023. Bars are coloured according to the WHO region of the country of notification, African regional office (AFRO, dark blue), Americas Regional Office AMRO, red) Eastern Mediterranean regional office (EMRO, purple), European regional office (EURO - light blue), Southeast Asian Regional Office (SEARO, dark red), Western Pacific regional office (WPRO, beige) and ordered by median maximum distance.

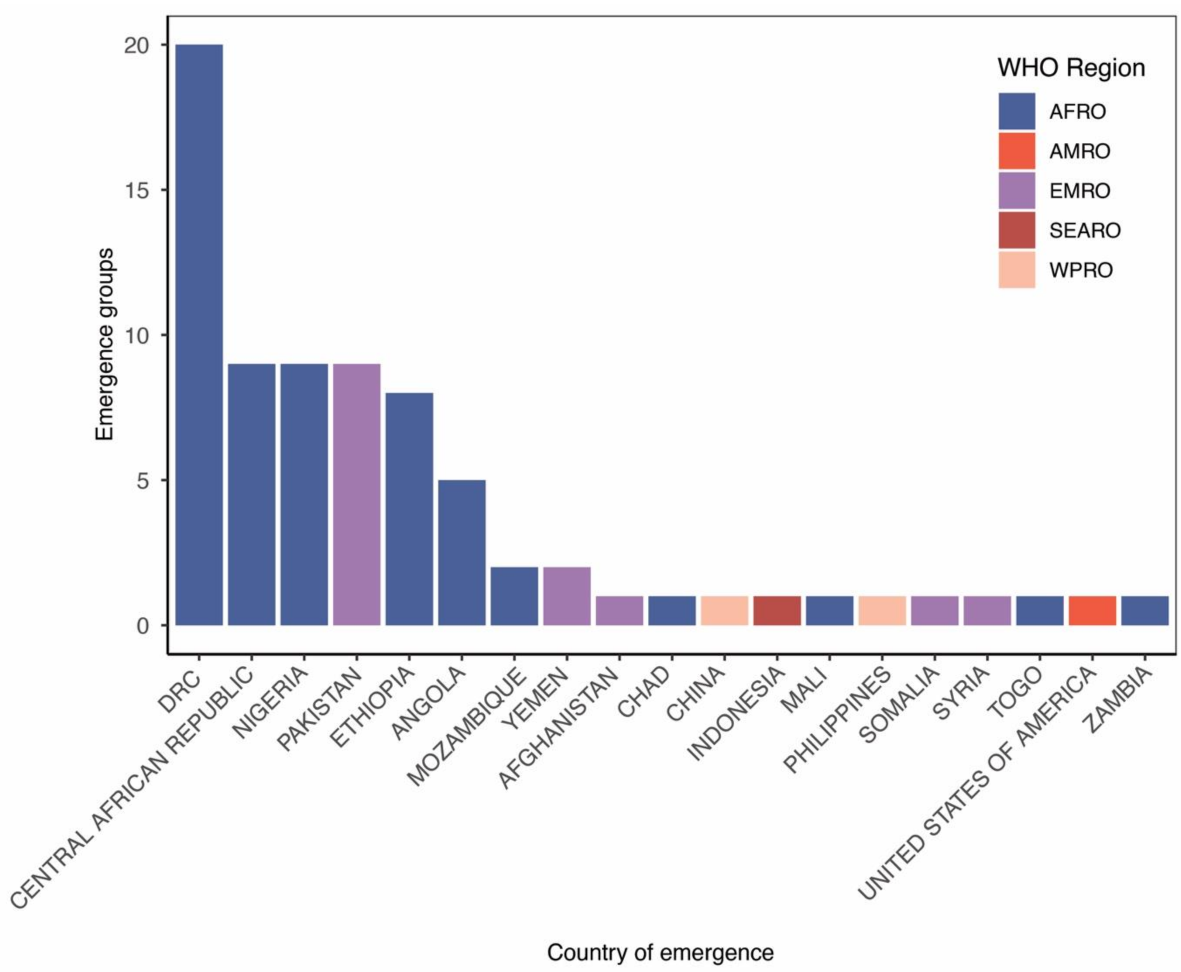

Fig. S3.
Country of emergence of 74 cVDPV2 outbreaks (emergence groups). Country of emergence was determined as the country where the first AFP case linked to that outbreak was reported. Bars are coloured according to the WHO region of the country of emergence, African regional office (AFRO, dark blue), Americas Regional Office AMRO, red) Eastern Mediterranean regional office (EMRO, purple), European regional office (EURO - light blue), Southeast Asian Regional Office (SEARO, dark red), Western Pacific regional office (WPRO, beige) and ordered by median maximum distance.

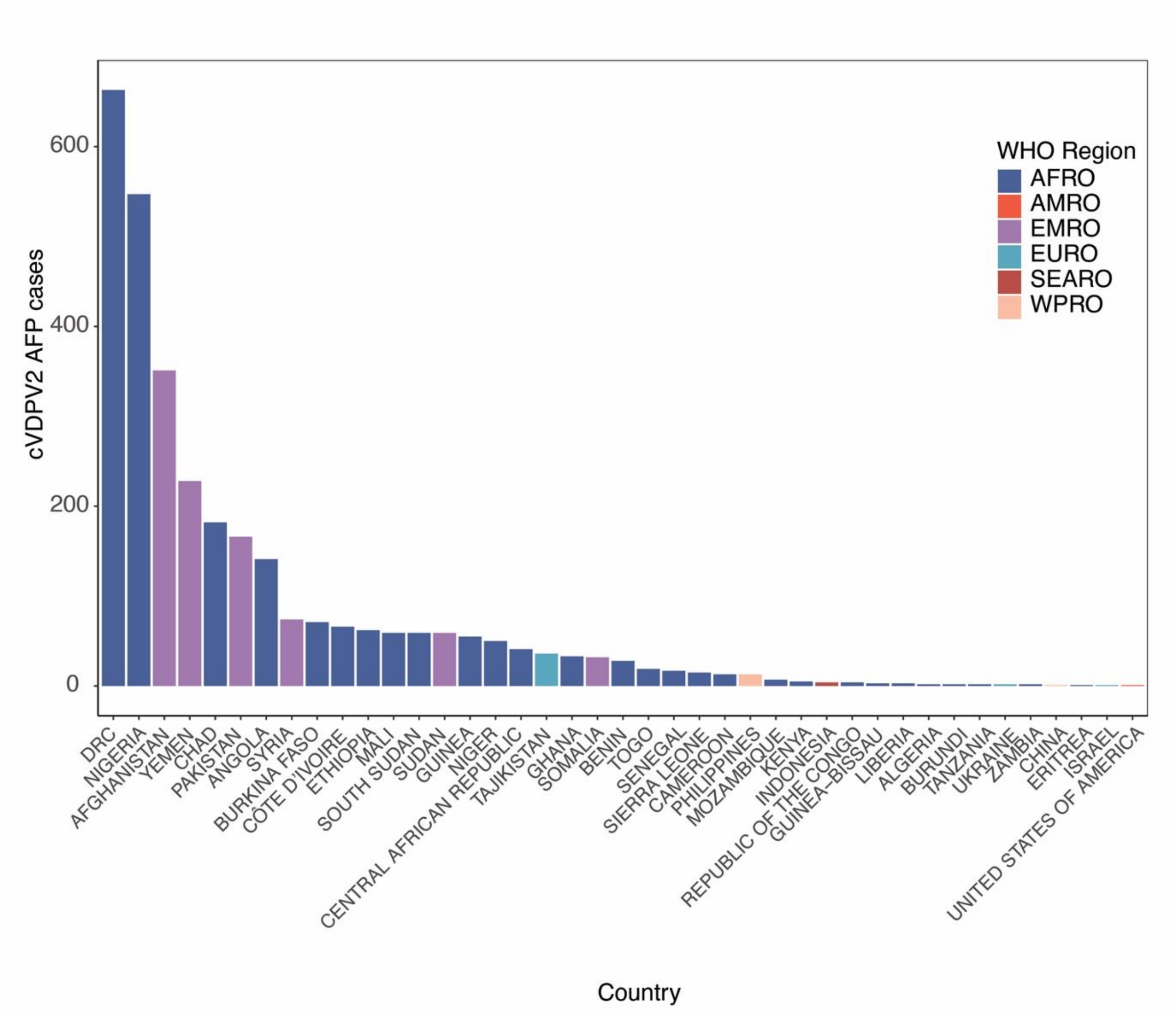

Fig. S4.
Distribution of cVDPV2 AFP cases per country of notification between May 2016 and September 2023. Bars are coloured according to the WHO region of the country of emergence, African regional office (AFRO, dark blue), Americas Regional Office AMRO, red) Eastern Mediterranean regional office (EMRO, purple), European regional office (EURO - light blue), Southeast Asian Regional Office (SEARO, dark red), Western Pacific regional office (WPRO, beige) and ordered by median maximum distance.

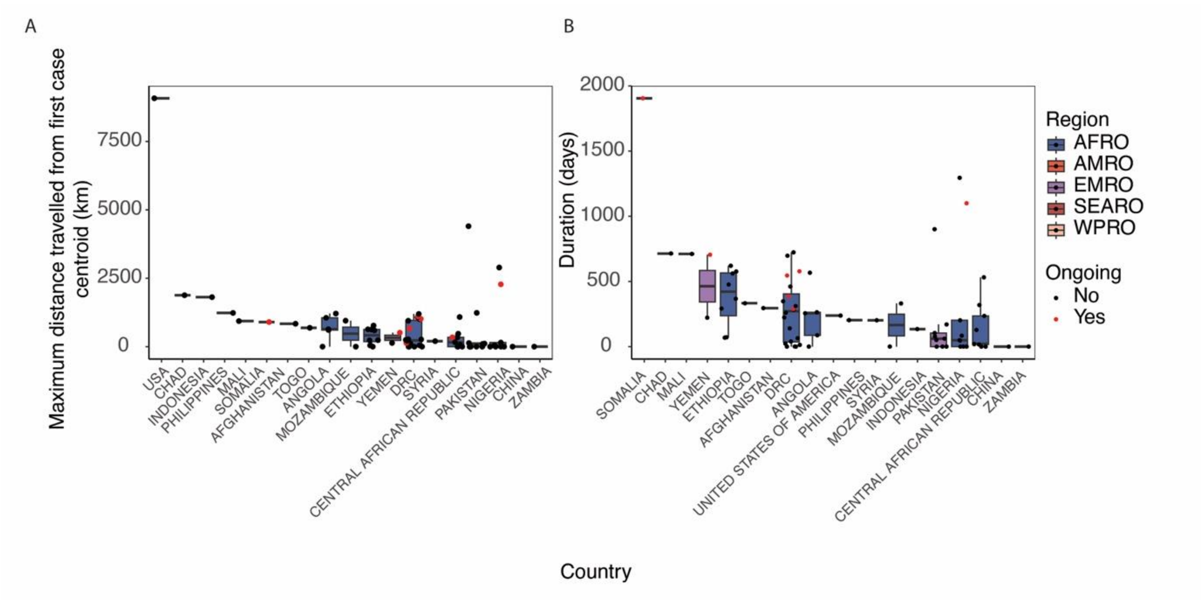

Fig. S5.
Maximum distance and duration of cVDPV2 outbreaks per country of emergence. A) Maximum distance travelled from outbreak origin for each outbreak by country of origin. B) Maximum outbreak duration for each outbreak by country of origin and ordered by median maximum duration. Points are coloured according to the WHO region of the country of emergence, African regional office (AFRO, dark blue), Americas Regional Office AMRO, red) Eastern Mediterranean regional office (EMRO, purple), European regional office (EURO - light blue), Southeast Asian Regional Office (SEARO, dark red), Western Pacific regional office (WPRO, beige) and ordered by median maximum distance. The central horizontal line indicates the median (50th percentile). The bounds of each box represent the interquartile range (IQR, 25th–75th percentiles). Whiskers extend to the most extreme data points within 1.5 × IQR from the lower and upper quartiles. Whisker ends correspond to the minimum and maximum non-outlier values.

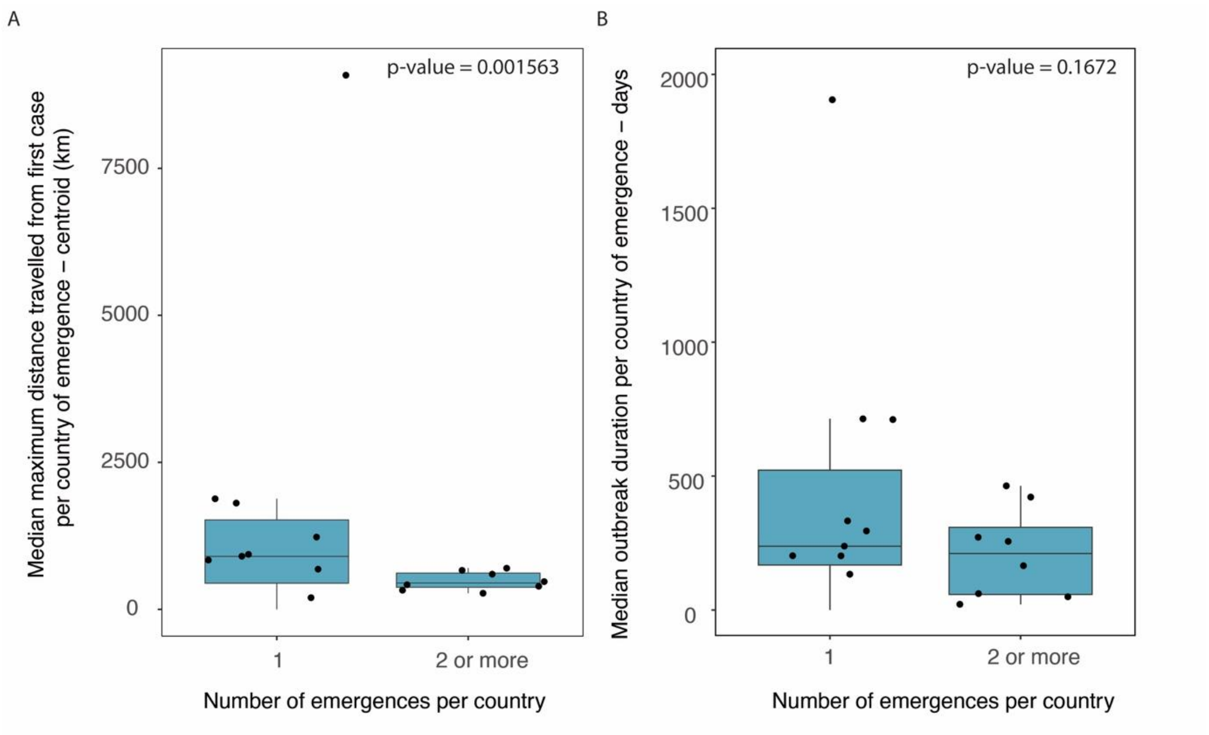

Fig. S6.
(A) Maximum distance of spread (km) in countries with single vs countries with multiple emergences of cVDPV2 outbreaks. For countries with multiple outbreaks, median maximum distance of spread was estimated considering all outbreaks emerged in the same country. (B) Maximum duration (days) in countries with single vs countries with multiple emergences of cVDPV2 outbreaks. For countries with multiple outbreaks, median maximum duration of spread was estimated considering all outbreaks emerged in the same country. The central horizontal line indicates the median (50th percentile). The bounds of each box represent the interquartile range (IQR, 25th–75th percentiles). Whiskers extend to the most extreme data points within 1.5 × IQR from the lower and upper quartiles. Whisker ends correspond to the minimum and maximum non-outlier values. Significance was tested using a two-tailed Mann-Whitney U test.

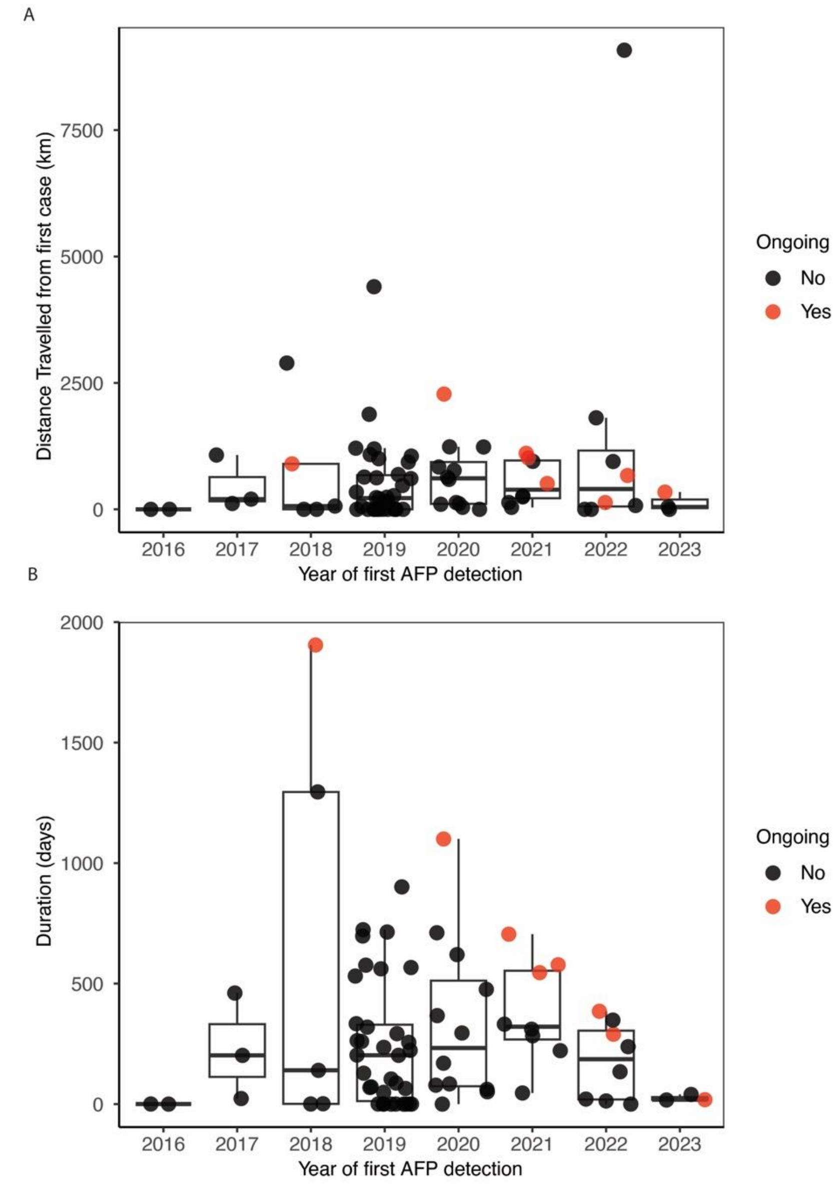

Fig. S7.
Maximum distance of spread (A) and maximum duration of spread (B) per outbreak per year of outbreak onset. Points are coloured according to whether the outbreak was still ongoing or not. Ongoing outbreaks were defined was those with cases in the previous six months at the time of data download (September 2023). The central horizontal line indicates the median (50th percentile). The bounds of each box represent the interquartile range (IQR, 25th–75th percentiles). Whiskers extend to the most extreme data points within 1.5 × IQR from the lower and upper quartiles. Whisker ends correspond to the minimum and maximum non-outlier values.

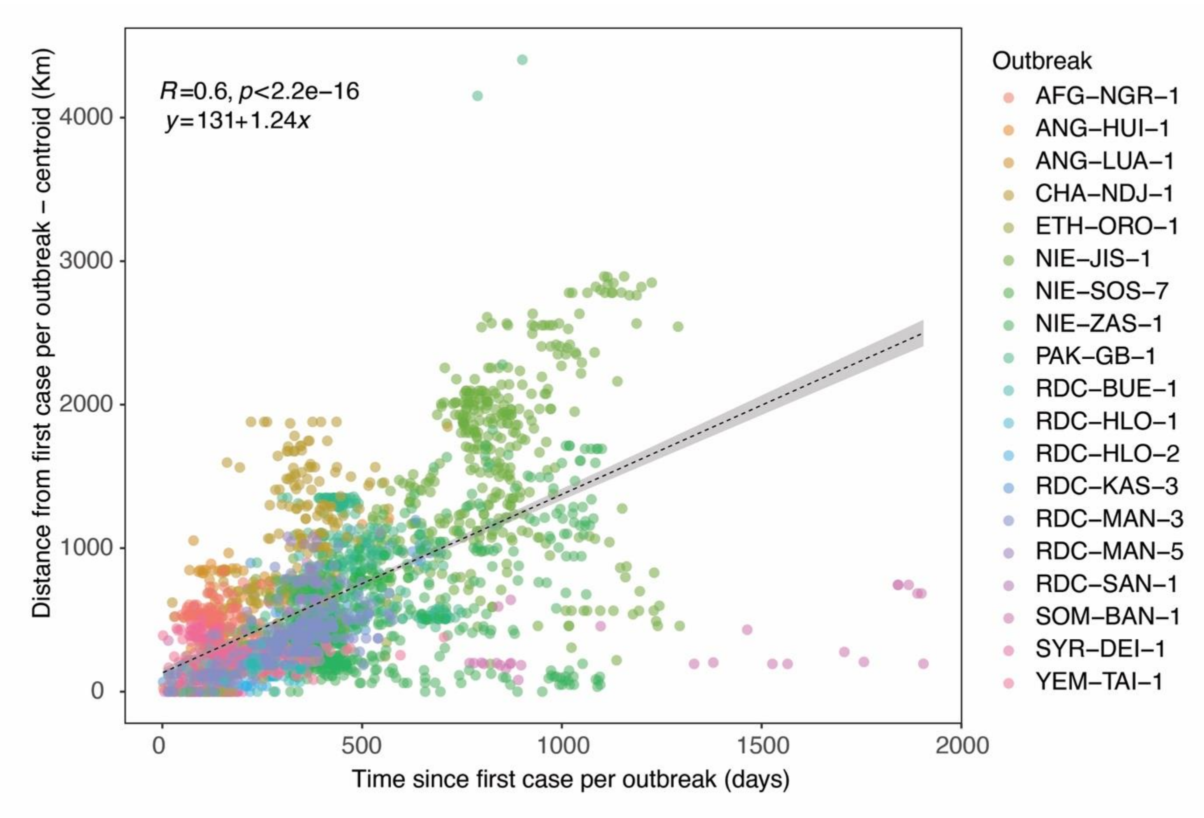

Fig. S8.
Correlation between distance and duration of spread for 19 large cVDPV2 outbreaks (>=20 polio AFP cases). Points are coloured according to outbreak. Text shows Pearson's r correlation and linear regression formula. Slope can also be interpreted as speed of spread when considering all cases in the outbreak. Dotted line and shaded area represent the estimated linear regression line and the 95% confidence interval (two-sided test).

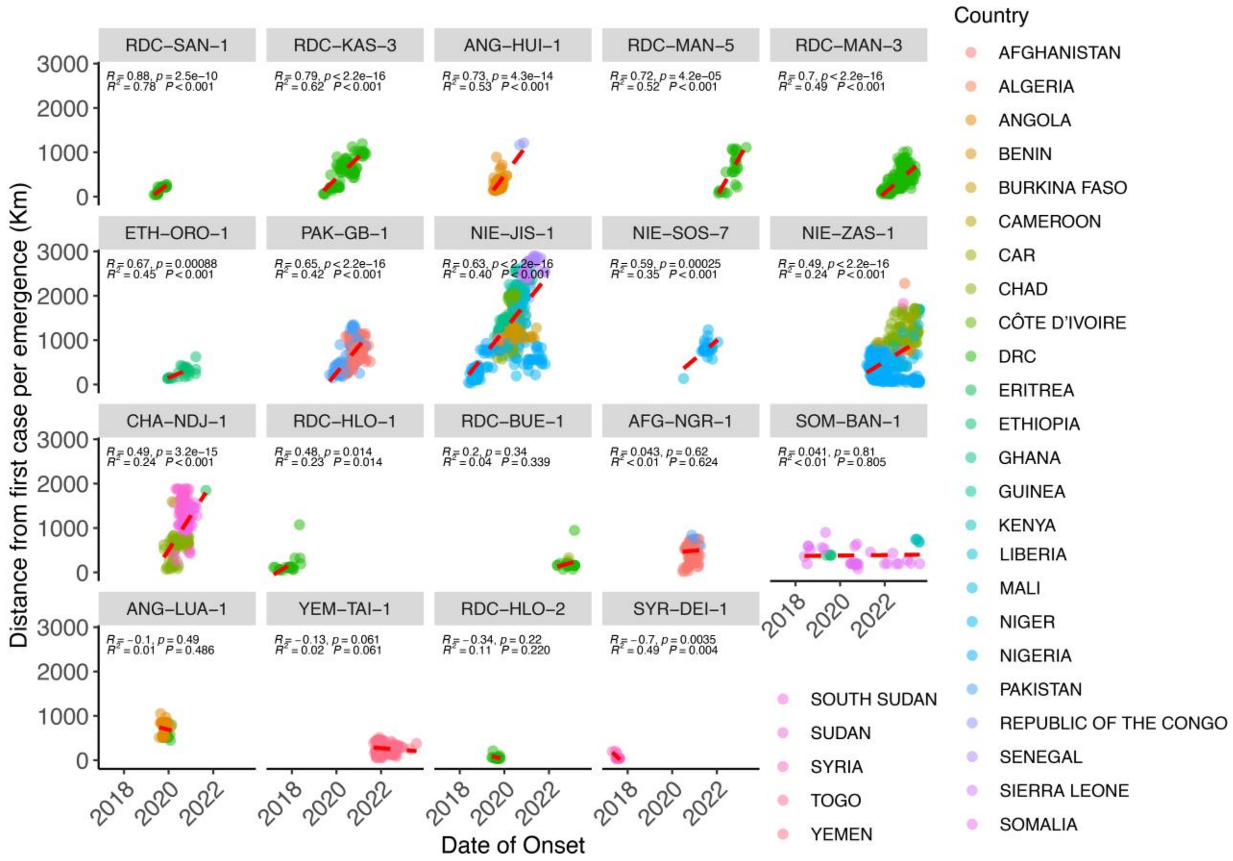

Fig. S9.

Correlation between distance and duration of spread for 19 large cVDPV2 outbreaks (>=20 polio AFP cases). Points are coloured according to country of notification. Text shows Pearson's r correlation, linear regression statistics an equation. Slope can also be interpreted as speed of spread when considering all cases in the outbreak. Dotted line represents the estimated linear regression line (two-sided test). Correlation was assessed using a two-sided Pearson correlation test. Sample size for each outbreak: NIE-ZAS-1 (n=578), NIE-JIS-1 (428), PAK-GB-1 (393), RDC-MAN-3 (340), YEM-TAI-1 (221), CHA-NDJ-1 (220), AFG-NGR-1 (135), RDC-KAS-3 (105), ANG-HUI-1 (79), SYR-DEI-1 (74), ANG-LUA-1 (49), SOM-BAN-1 (40), NIE-SOS-7 (35), RDC-SAN-1 (32), ETH-ORO-1 (28), RDC-HLO-1 (27), RDC-MAN-5 (26), RDC-BUE-1 (24), RDC-HLO-2 (20).

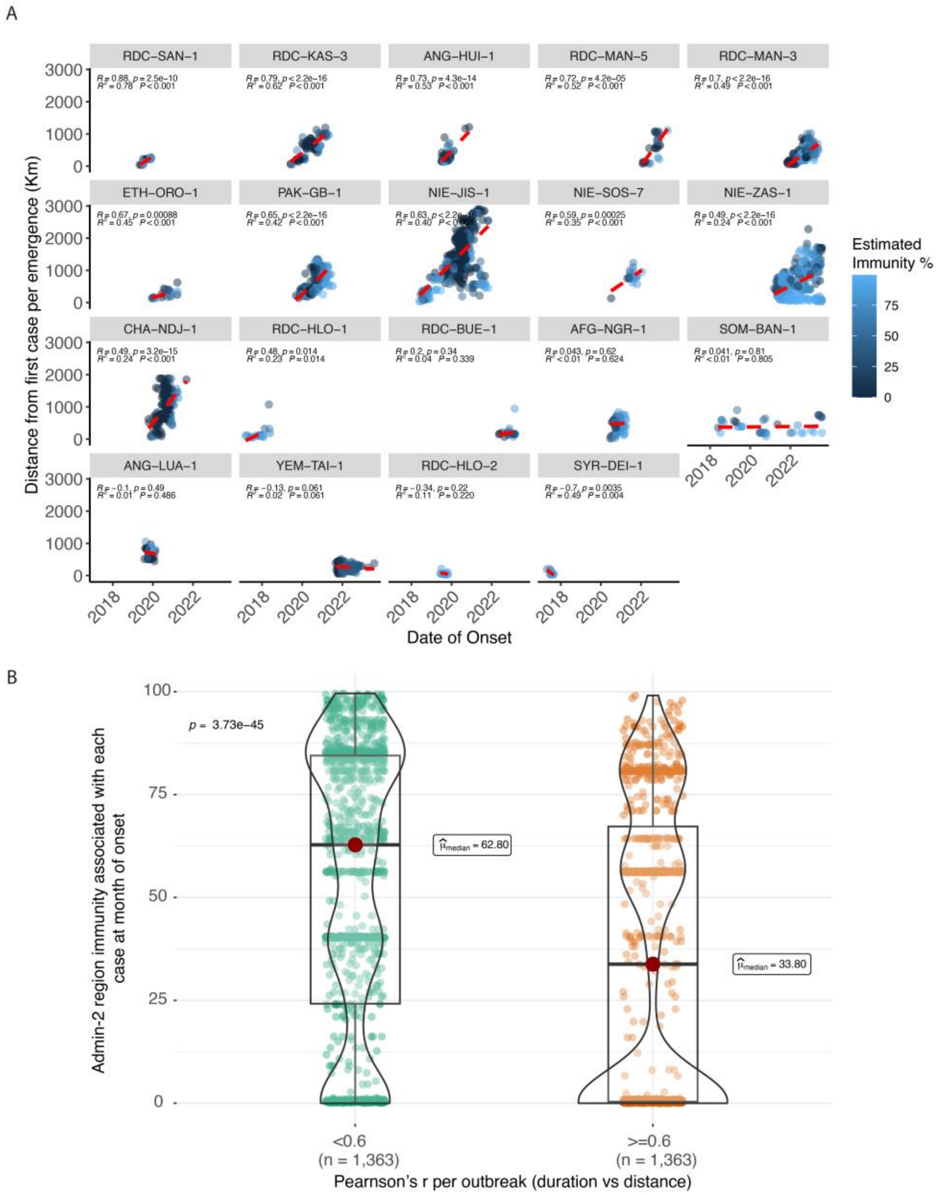

Fig. S10.

Relationship between distance and duration correlation and levels of administrative region 2 immunity at the time of case onset. (A) Correlation between distance and duration of spread for 19 large cVDPV2 outbreak (>= 20 polio AFP cases). Points are coloured according to estimated type-2 population immunity in children 6-36 months of age at the admin 2 level of notification in the same month of case symptom onset. Text shows Spearman's correlation coefficient, linear regression statistics. Sample size for each outbreak: NIE-ZAS-1 (n=578), NIE-JIS-1 (428), PAK-GB-1 (393), RDC-MAN-3 (340), YEM-TAI-1 (221), CHA-NDJ-1 (220), AFG-NGR-1 (135), RDC-KAS-3 (105), ANG-HUI-1 (79), SYR-DEI-1 (74), ANG-LUA-1 (49), SOM-BAN-1 (40), NIE-SOS-7 (35), RDC-SAN-1 (32), ETH-ORO-1 (28), RDC-HLO-1 (27), RDC-MAN-5 (26), RDC-BUE-1 (24), RDC-HLO-2 (20). (B) Comparison between the estimated admin-2-level immunity for outbreaks with strong correlation between duration and distance (Pearson's r >=0.6) vs other outbreaks (Pearson's r <0.6). Cases and their respective admin-2 immunity at the time of symptom onset were discretized according to the level of correlation between distance and duration (time of detection) for the outbreak (emergence group) they are linked to (see Figure S10A). The central horizontal line indicates the median (50th percentile). The bounds of each box represent the interquartile range (IQR, 25th–75th percentiles). Whiskers extend to the most extreme data points within 1.5 × IQR from the lower and upper quartiles. Whisker ends correspond to the minimum and maximum non-outlier values.

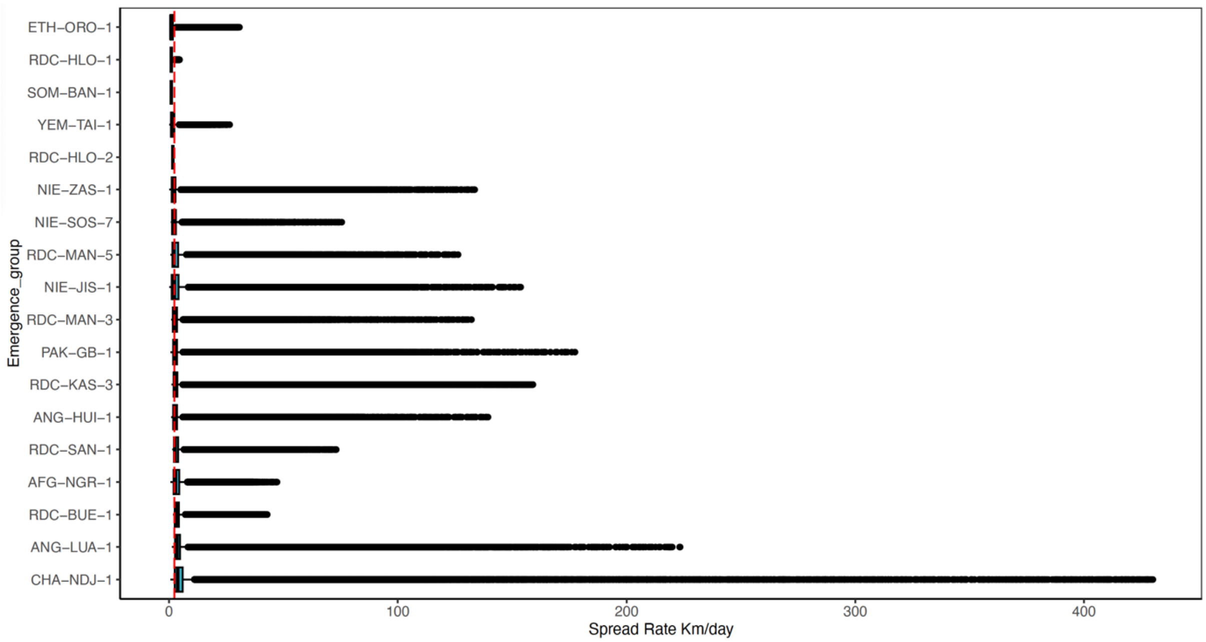

Fig. S11.
Full version of Figure 3B including outliers. Box plot showing the distribution of wavefront velocity estimates for large cVDPV2 outbreak. The central horizontal line indicates the median (50th percentile). Sample size per outbreak at the start of the analysis: NIE-ZAS-1 (n=578), NIE-JIS-1 (428), PAK-GB-1 (393), RDC-MAN-3 (340), YEM-TAI-1 (221), CHA-NDJ-1 (220), AFG-NGR-1 (135), RDC-KAS-3 (105), ANG-HUI-1 (79), ANG-LUA-1 (49), SOM-BAN-1 (40), NIE-SOS-7 (35), RDC-SAN-1 (32), ETH-ORO-1 (28), RDC-HLO-1 (27), RDC-MAN-5 (26), RDC-BUE-1 (24), RDC-HLO-2 (20). The bounds of each box represent the interquartile range (IQR, 25th–75th percentiles). Whiskers extend to the most extreme data points within 1.5 × IQR from the lower and upper quartiles. Whisker ends correspond to the minimum and maximum non-outlier values.

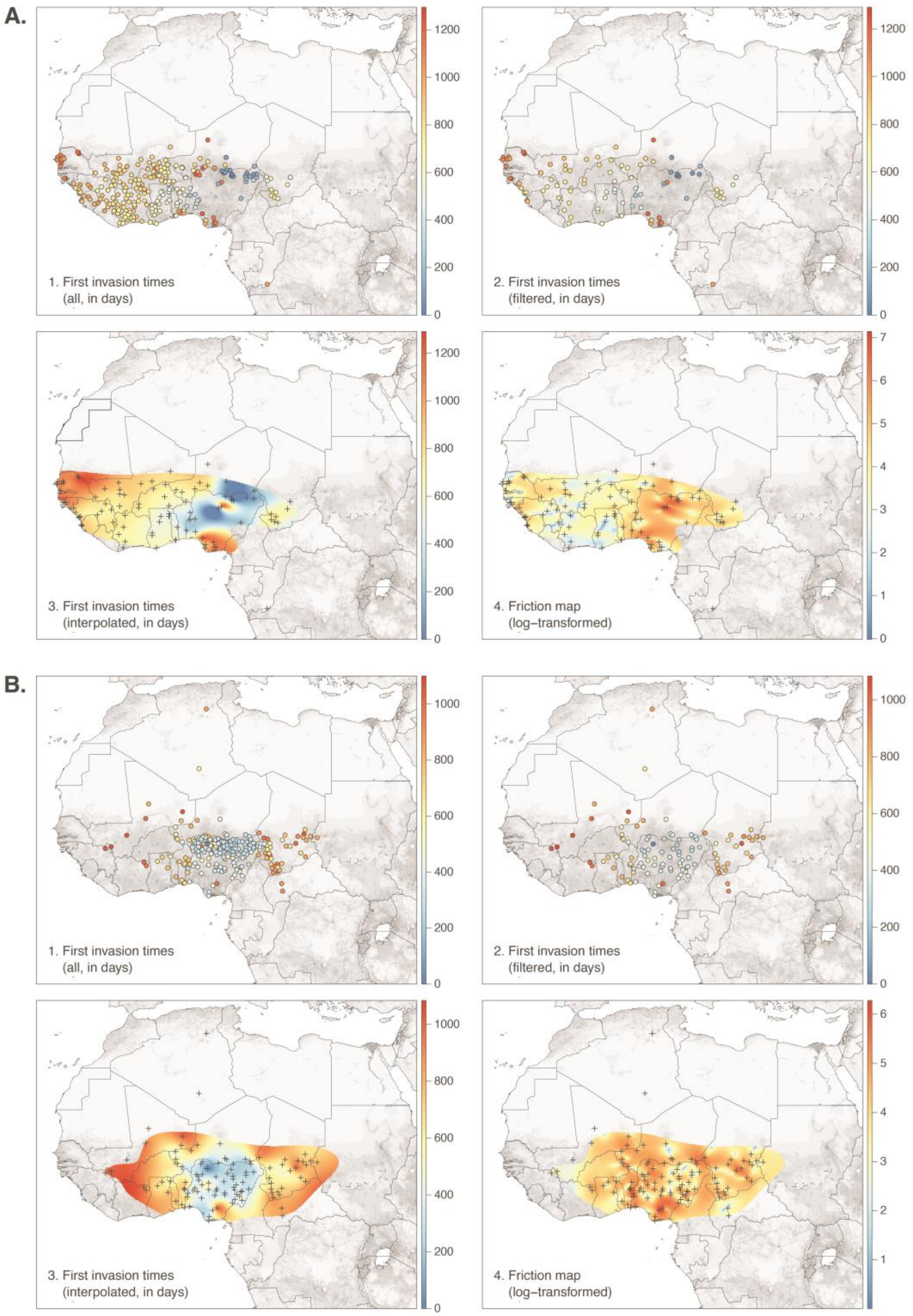

Fig. S12.
Example of wavefront velocity analysis workflow using the (A) NIE-JIS-1(n= 578) and the (B) NIE-ZAS-1 (n=428) outbreaks as examples. (1) Invasion times (days) for all cVDPV2 AFP cases reported. Invasion times were calculated as the difference in days between the onset of each case and the first case of the outbreak (origin). (2) First invasion times for filtered cases only. Cases were filtered to include only those extending the wavefront of the outbreak (see methods). (3) Interpolation of invasion times for filtered cases on the raster surface. (4) Estimation of friction (time/distance) based on the interpolated invasion times for filtered cases. Outbreak wavefront velocity was estimated as the inverse of the friction (not shown, see Methods for details).

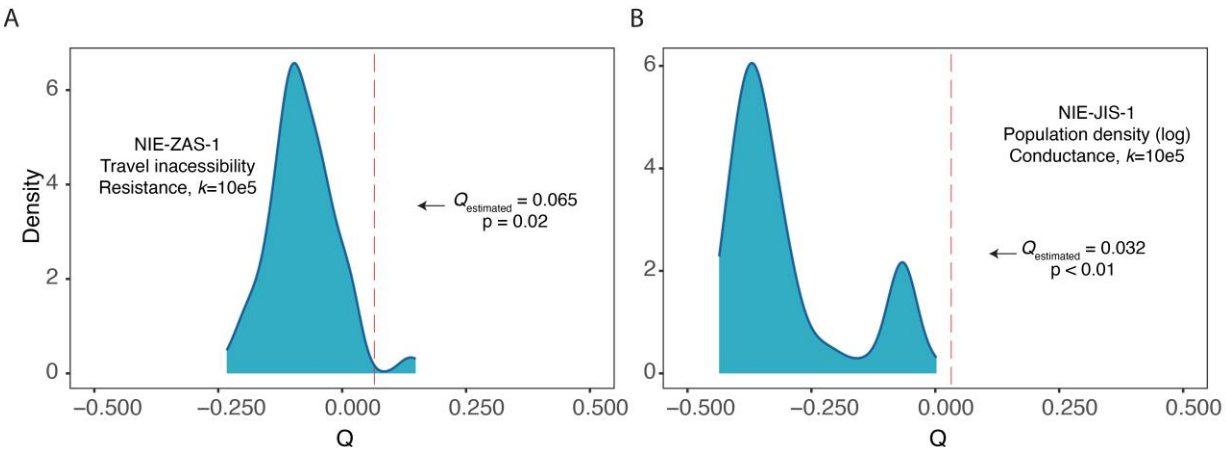

Fig. S13.
Additional factors significantly impacting the wavefront velocity of the two largest cVDPV2 outbreaks to date, NIE-ZAS-1 (578, ongoing) and NIE-JIS-1 (428). A) Impact of travel inaccessibility (minutes to travel one meter) in the wavefront velocity of the NIE-ZAS-1 outbreak and B) Impact of population density in the wavefront velocity of the NIE-JIS-1 outbreak. Red dotted lines represent the actual estimated *Q* for each outbreak. *Q* represents the proportion of the heterogeneity in the wavefront velocity that can be associated with the tested variable (see the Supplementary Materials for full details). Density plots depict the distribution of *Q* values obtained under a null dispersal model in which the tested factors do not impact the outbreak wavefront velocity. As detailed in Supplementary Materials, such a null dispersal model being obtained by a stochastic rotation of dispersal vectors.

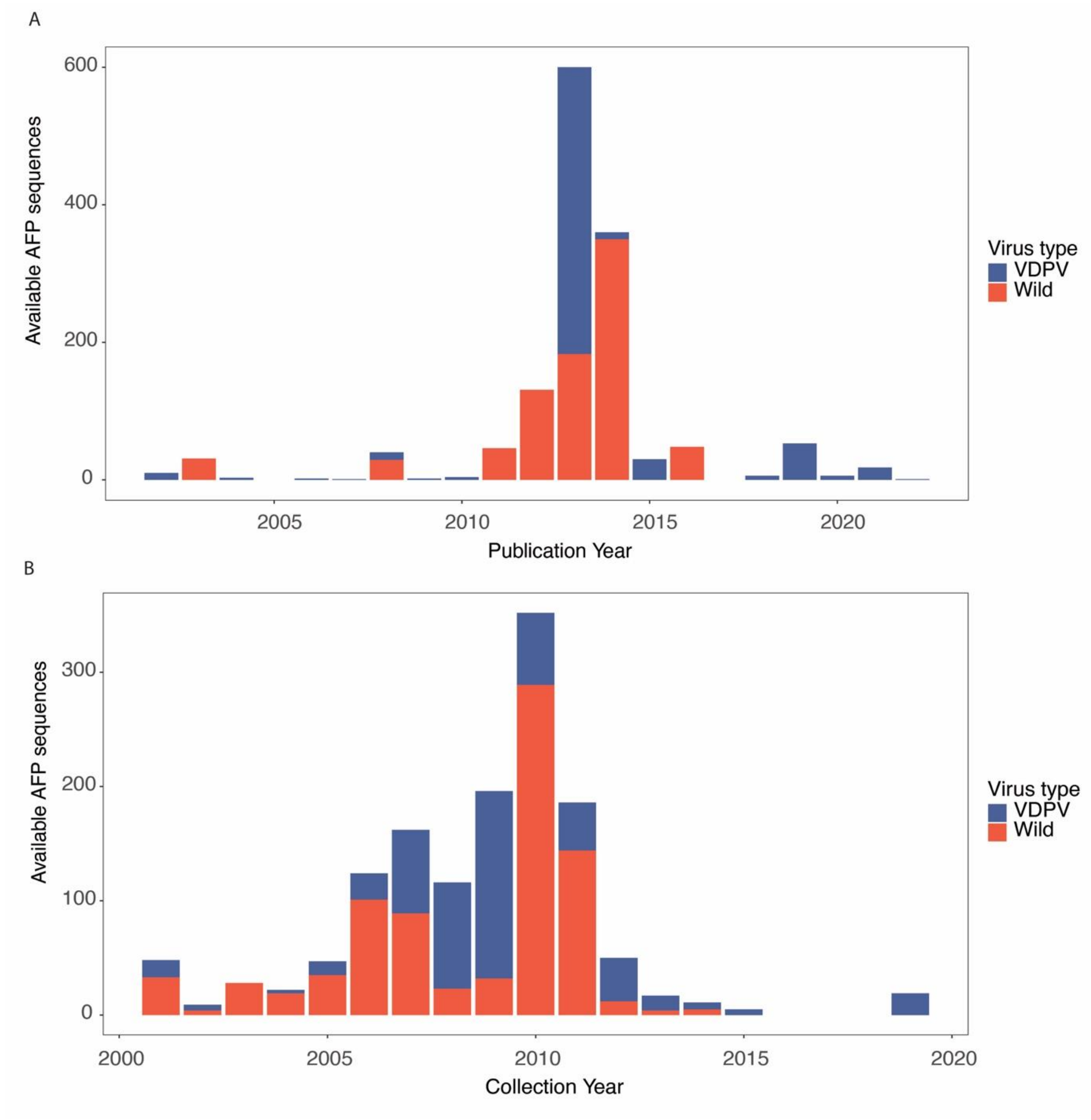

Fig. S14.
Availability of VDPV (blue) and WPV (red) AFP sequences on GenBank according to publication year (A) and collection year (B). Sequences and metadata were downloaded in July 2022. Sample source (AFP) was identified according to GenBank metadata or to publications linked to the sequences.

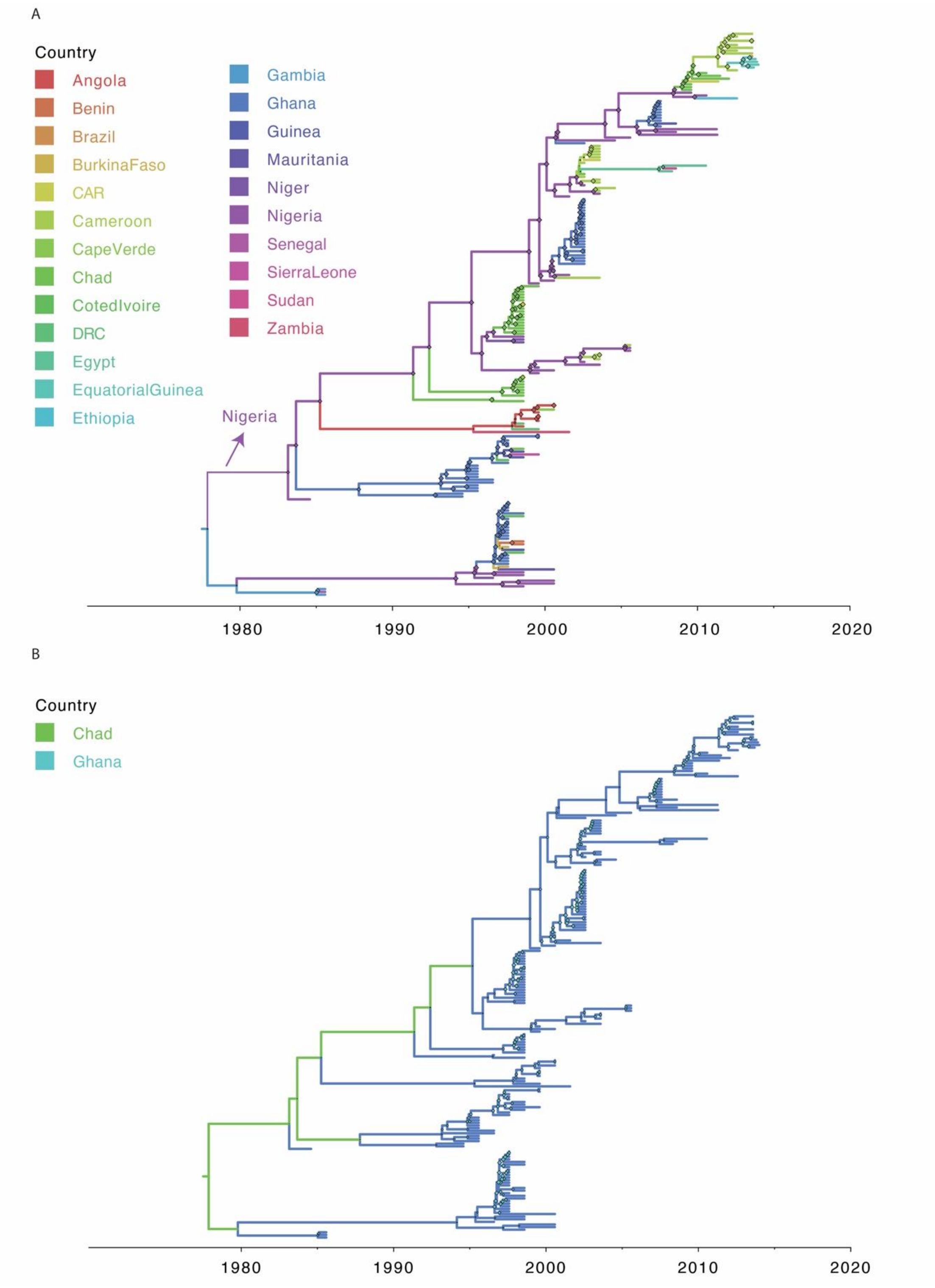

Fig. S15.
Phylogeographic reconstruction of WPV1 sequences in Clade 2 (n=201). Maximum clade credibility phylogenies represent a regular discrete trait analysis (DTA) at the country of collection level (A) and a DTA using a tip-swapping approach (see methods) for investigation of sampling bias and transition rates correction. Tree branches are coloured according to the location inferred through DTA. Diamonds are coloured according to inferred location and are sized according to location probability.

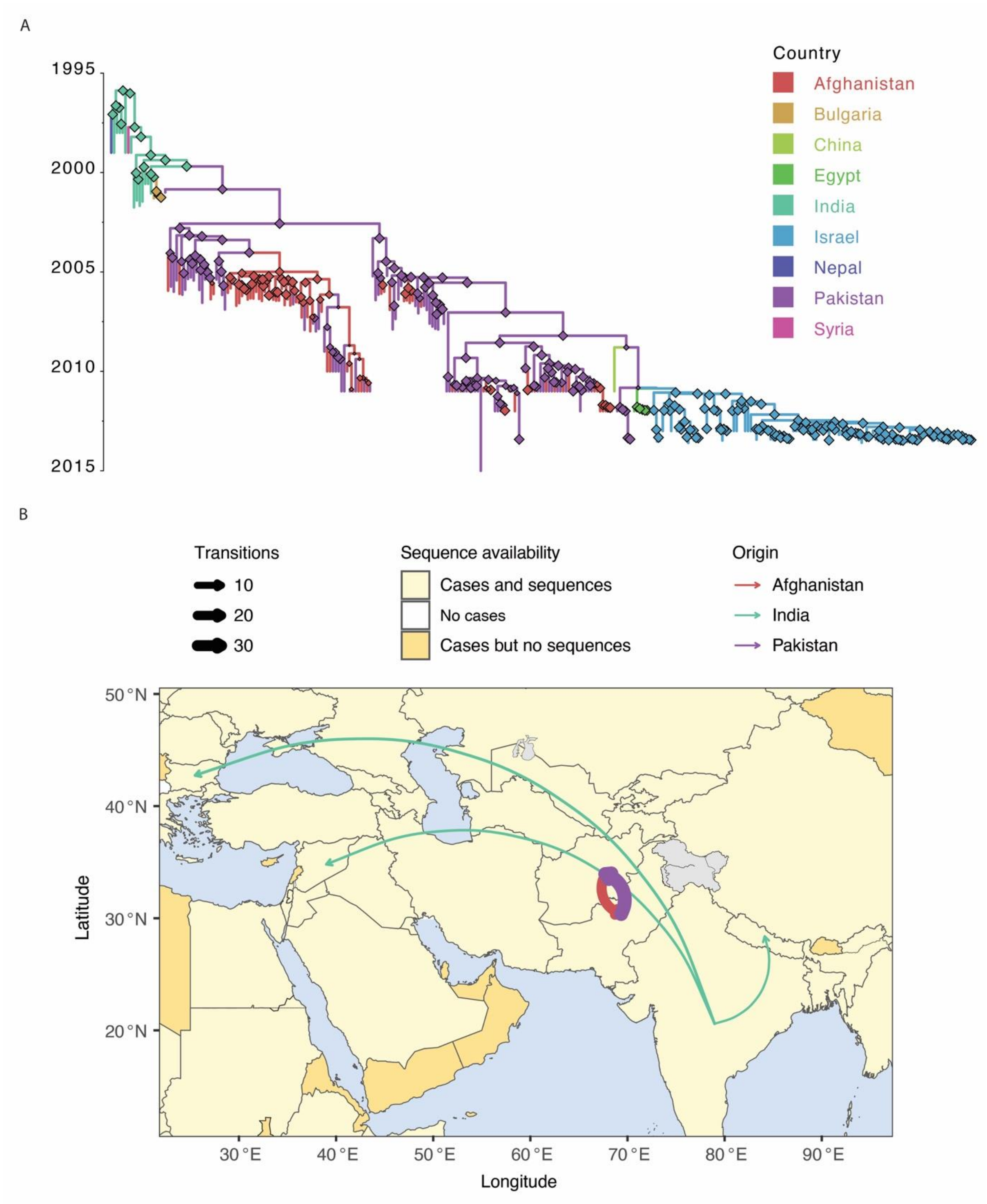

Fig. S16.
Discrete phylogeographic analysis of 304 historical WPV1 VP1 sequences from Clade 4a (see methods). (A) Time-rooted phylogeny of 304 sequences from cases and environmental samples downloaded from GenBank covering the period between 1998-2015. Location was discretised according to country of collection. **(B)** Sequence availability map and well-supported movements (Markov jumps, adjusted Bayes Factor > 20). Countries are coloured according to reporting of cases and availability of sequences: countries that reported cases and sequences (light yellow), countries with cases but no sequences available (dark yellow).

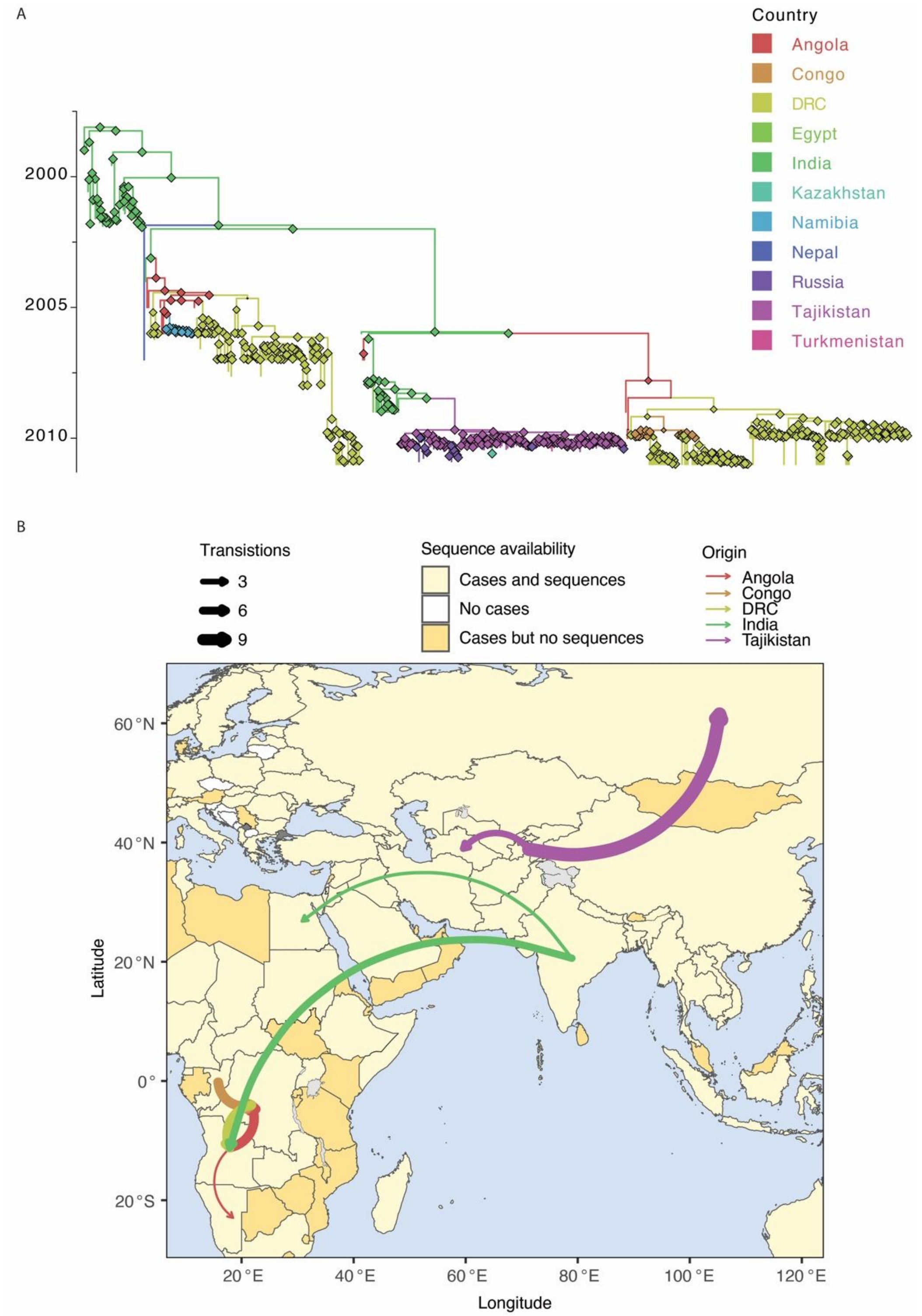

Fig. S17.
Discrete phylogeographic analysis of 559 historical WPV1 VP1 sequences from Clade 4b (see methods). (A) Time-rooted phylogeny of 559 sequences from cases and environmental samples downloaded from GenBank covering the period between 1999-2015. Location was discretised according to country of collection. **(B)** Sequence availability map and well-supported movements (Markov jumps, adjusted Bayes Factor > 20). Countries are coloured according to reporting of cases and availability of sequences: countries that reported cases and sequences (light yellow), countries with cases but no sequences available (dark yellow).

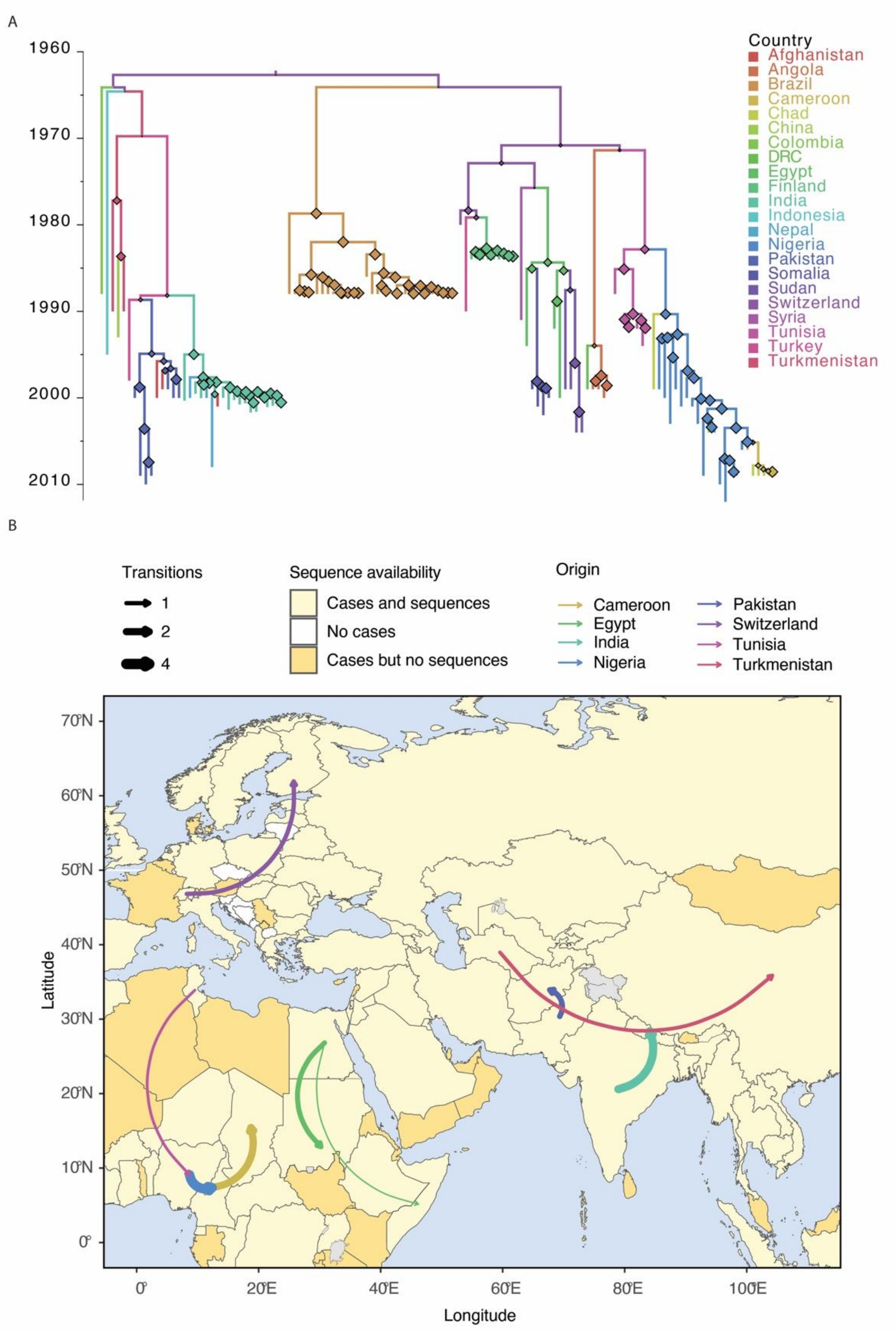

Fig. S18.
Discrete phylogeographic analysis of 123 historical WPV3 VP1 sequences (see methods). (A) Time-rooted phylogeny of 123 sequences from cases and environmental samples downloaded from GenBank covering the period between 1980-2012. Location was discretised according to country of collection. **(B)** Sequence availability map and well-supported movements (Markov jumps, adjusted Bayes Factor > 20). Countries are coloured according to reporting of cases and availability of sequences: countries that reported cases and sequences (light yellow), countries with cases but no sequences available (dark yellow).

Fig. S19.

Full version of phylogeny presented in Fig 4A. Discrete phylogeographic analysis of 1572 historical WPV1 VP1 sequences. (A) Time-rooted phylogeny of 1572 sequences from cases and environmental samples downloaded from GenBank covering the period between 1958-2015. Country of collection was discretised according to 13 global regions and clades were identified based on the posterior node support and location support for internal nodes (see Methods).

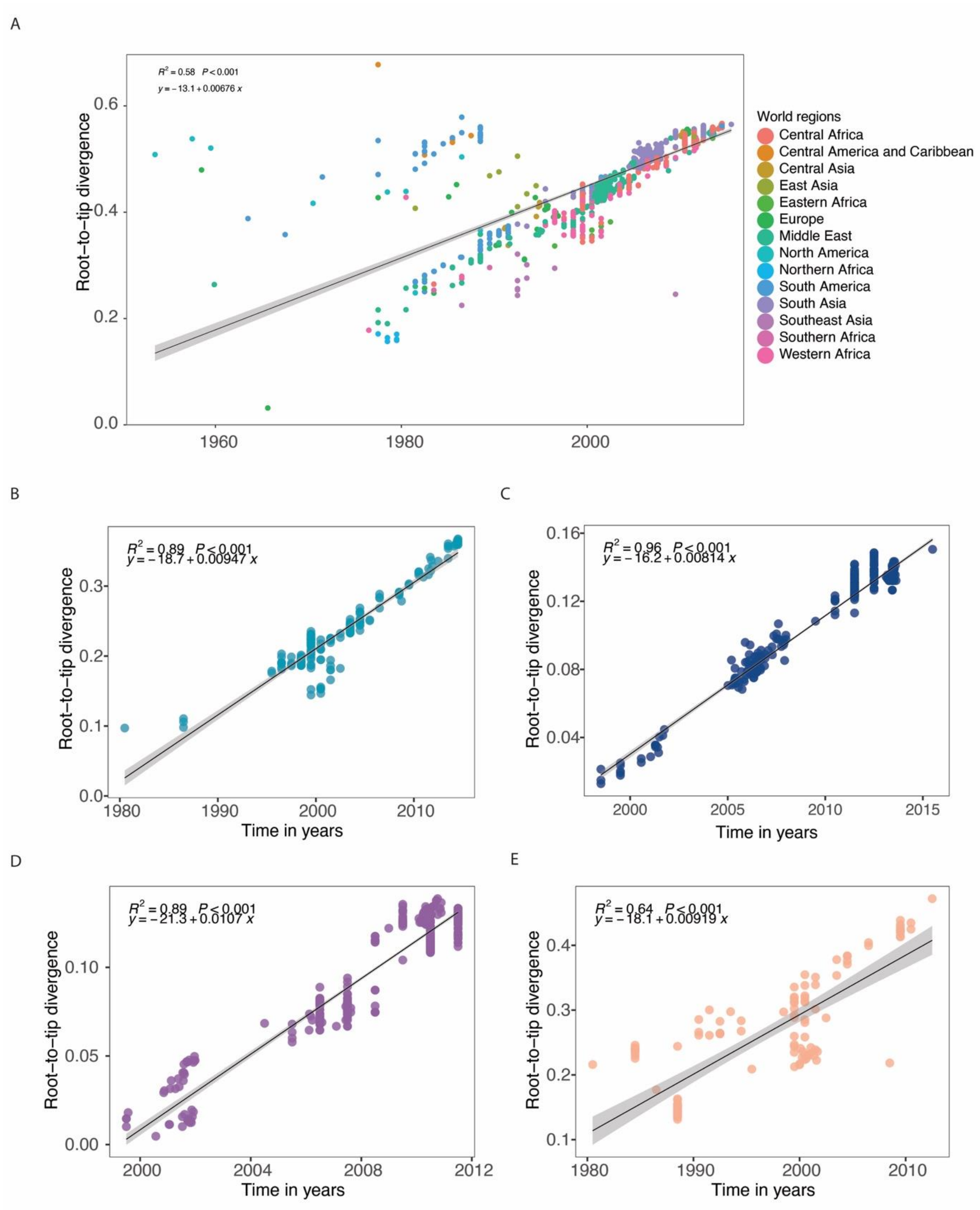

Fig.

Fig. S20.
Regression of root-to-tip genetic diversity against collection date for poliovirus sequences in datasets A (global analysis, n=1572, A), B (Clade 2, n= 201, B), C (Clade 4a, n= 304, C), D (Clade 4B, n= 5539, D) and E (WPV3, n= 123, E). Dotted line and shaded area represent the estimated linear regression line and the 95% confidence interval (two-sided test). Correlation was assessed using a two-sided Pearson correlation test.

Table S1. **Metadata on each cVDPV2 outbreak included in the period of analysis,** May 1, 2016, and September 29, 2023**.**

Outbreak	Country of 1st reported AFP case	Total number of reported Cases	Outbreak Duration (days)	Year of outbreak detection	Countries with reported AFP cases
AFG-HLD-1*	Afghanistan	6	170	2020	Afghanistan, Pakistan
AFG-NGR-1	Afghanistan	135	295	2020	Afghanistan, Pakistan
ANG-HUI-1	Angola	79	567	2019	Angola, Republic of The Congo
ANG-LNO-1	Angola	1	0	2019	Angola
ANG-LNO-2	Angola	17	263	2019	Angola, DRC
ANG-LUA-1	Angola	49	256	2019	Angola, DRC
ANG-MOX-1	Angola	13	87	2019	Angola, Zambia
CAF-BAM-1	CAR	5	128	2019	CAR
CAF-BER-1	CAR	5	319	2019	Cameroon, CAR
CAF-BIM-1	CAR	1	0	2019	CAR
CAF-BIM-3	CAR	4	531	2019	Chad, CAR
CAF-BNG-1	CAR	12	235	2019	Chad, CAR
CAF-BNG-2	CAR	3	21	2022	CAR
CAF-BNG-3	CAR	3	18	2023	CAR
CAF-KEM-1	CAR	1	0	2022	CAR
CAF-MOZ-1	CAR	3	17	2023	CAR
CHA-NDJ-1	Cameroon	220	714	2019	Chad, Sudan, South Sudan, Cameroon, Eritrea, CAR
CHN-SIC-1	China	1	0	2019	China
ETH-ORO-1	Ethiopia	28	561	2019	Ethiopia
ETH-ORO-2	Ethiopia	4	71	2019	Ethiopia
ETH-ORO-3	Ethiopia	2	292	2019	Ethiopia
ETH-ORO-4	Ethiopia	2	69	2019	Ethiopia
ETH-SOU-1	Ethiopia	11	620	2020	South Sudan, Ethiopia
ETH-SOU-2	Ethiopia	7	576	2019	Ethiopia
ETH-SOU-3	Ethiopia	2	476	2020	Ethiopia
INO-ACE-1	Indonesia	4	134	2022	Indonesia
IUUC-2022	Usa	2	238	2022	Israel, USA
MOZ-NPL-1	Mozambique	6	331	2021	Mozambique
MOZ-ZAM-2	Mozambique	1	0	2018	Mozambique
NIE-JIS-1	Nigeria	428	1295	2018	Nigeria, Côte D'Ivoire, Niger, Ghana, Benin, Mali, Burkina Faso, Guinea-Bissau, Guinea, Liberia, Republic of The Congo, Chad, Senegal, Sierra Leone, Togo

NIE-KBS-1	Nigeria	2	46	2021	Nigeria
NIE-KGS-1	Nigeria	3	202	2019	Nigeria
NIE-KGS-2	Nigeria	2	49	2019	Nigeria
NIE-SOS-2	Nigeria	1	0	2016	Nigeria
NIE-SOS-3	Nigeria	1	0	2019	Nigeria
NIE-SOS-5	Nigeria	1	0	2019	Nigeria
NIE-SOS-7	Nigeria	35	711	2020	Nigeria, Niger, Mali
NIE-SOS-8	Nigeria	2	84	2020	Nigeria
NIE-ZAS-1	Nigeria	578	1100	2020	Nigeria, Niger, Benin, Chad, Côte D’Ivoire, Cameroon, Guinea, Mali, Ghana, Algeria, Togo, CAR, Sudan
PAK-FSD-1	Pakistan	11	61	2020	Pakistan
PAK-FSD-2	Pakistan	2	51	2020	Pakistan
PAK-GB-1	Pakistan	393	901	2019	Afghanistan, Pakistan, Tajikistan, Ukraine
PAK-GB-3	Pakistan	1	0	2019	Pakistan
PAK-KOH-1	Pakistan	1	0	2019	Pakistan
PAK-LKW-1	Pakistan	3	78	2020	Pakistan
PAK-QTA-1	Pakistan	1	0	2016	Pakistan
PAK-TOR-1	Pakistan	2	104	2019	Pakistan
PHL-NCR-1	Philippines	13	203	2019	Philippines
RDC-BUE-1	DRC	24	348	2022	DRC, CAR
RDC-EQT-1	DRC	1	0	2020	DRC
RDC-HKA-1	DRC	2	1	2018	DRC
RDC-HLO-1	DRC	27	461	2017	DRC
RDC-HLO-2	DRC	20	260	2019	DRC
RDC-KAS-1	DRC	2	723	2019	DRC, Republic of The Congo
RDC-KAS-2	DRC	4	65	2019	DRC
RDC-KAS-3	DRC	105	697	2019	DRC
RDC-KOR-1	DRC	9	40	2023	DRC
RDC-MAN-1	DRC	2	23	2017	DRC
RDC-MAN-2	DRC	5	283	2021	DRC
RDC-MAN-3	DRC	340	578	2021	DRC
RDC-MAN-4	DRC	11	311	2021	DRC
RDC-MAN-5	DRC	26	545	2021	DRC
RDC-MON-1	DRC	11	140	2018	DRC
RDC-SAN-1	DRC	32	223	2019	DRC
RDC-SKV-1	DRC	16	290	2022	Burundi, DRC, Tanzania
RDC-TAN-2	DRC	2	13	2022	DRC
RDC-TPA-1	DRC	1	0	2019	DRC
RDC-TSH-1	DRC	14	385	2022	DRC

SOM-AWL-1	Somalia	4	367	2020	Ethiopia, Somalia
SOM-BAN-1	Somalia	40	1905	2018	Somalia, Kenya, Ethiopia
SYR-DEI-1	Syria	74	202	2017	Syria
TOG-SAV-1	Togo	12	333	2019	Burkina Faso, Togo, Côte D'Ivoire
YEM-SAN-1	Yemen	7	222	2021	Yemen
YEM-TAI-1	Yemen	221	705	2021	Yemen
ZAM-LUA-1	Zambia	1	0	2019	Zambia

*Outbreak names are coded according to country of detection (administrative region 1, first 3 letters), province of detection (administrative region 2, second 3 letters) and number of outbreak detected in that country and province.
DRC= Democratic Republict of The Congo, CAR = Central African Republic and USA= United States of America.

Table S2.
Impact of demographic and environmental variables on the wavefront velocity of cVDPV2 outbreaks.

Outbreak	Variable	Impact type	*k*	*Q*	p-value
NIE-ZAS-1	Human pop. density (log)	Conductance	10	-0.039	NA
NIE-ZAS-1	Human pop. density (log)	Conductance	100	-0.021	NA
NIE-ZAS-1	Human pop. density (log)	Conductance	1000	-0.015	NA
NIE-ZAS-1	Human pop. density (log)	Conductance	10000	-0.014	NA
NIE-ZAS-1	Human pop. density (log)	Conductance	100000	-0.014	NA
NIE-ZAS-1	Travel inaccessibility	Resistance	10	0.003	0.52
NIE-ZAS-1	Travel inaccessibility	Resistance	100	0.020	0.18
NIE-ZAS-1	Travel inaccessibility	Resistance	1000	0.056	0.02
NIE-ZAS-1	Travel inaccessibility	Resistance	10000	0.064	0.02
NIE-ZAS-1	Travel inaccessibility	Resistance	100000	0.065	0.02
NIE-ZAS-1	International borders	Resistance	10	0.03	< 0.01
NIE-ZAS-1	International borders	Resistance	100	0.21	< 0.01
NIE-ZAS-1	International borders	Resistance	1000	0.382	< 0.01
NIE-ZAS-1	International borders	Resistance	10000	0.379	< 0.01
NIE-ZAS-1	International borders	Resistance	100000	0.374	< 0.01
NIE-ZAS-1	International borders	Conductance	10	-0.015	NA
NIE-ZAS-1	International borders	Conductance	100	-0.037	NA
NIE-ZAS-1	International borders	Conductance	1000	-0.033	NA
NIE-ZAS-1	International borders	Conductance	10000	-0.027	NA
NIE-ZAS-1	International borders	Conductance	100000	-0.026	NA
NIE-JIS-1	Human pop. density (log)	Conductance	10	0.028	0.09
NIE-JIS-1	Human pop. density (log)	Conductance	100	0.032	<0.01
NIE-JIS-1	Human pop. density (log)	Conductance	1000	0.032	< 0.01
NIE-JIS-1	Human pop. density (log)	Conductance	10000	0.032	< 0.01
NIE-JIS-1	Human pop. density (log)	Conductance	100000	0.032	< 0.01
NIE-JIS-1	Travel inaccessibility	Resistance	10	0.005	0.11
NIE-JIS-1	Travel inaccessibility	Resistance	100	0.029	0.14
NIE-JIS-1	Travel inaccessibility	Resistance	1000	0.045	0.17
NIE-JIS-1	Travel inaccessibility	Resistance	10000	0.041	0.17
NIE-JIS-1	Travel inaccessibility	Resistance	100000	0.040	0.17
NIE-JIS-1	International borders	Resistance	10	-0.133	NA
NIE-JIS-1	International borders	Resistance	100	-0.339	NA
NIE-JIS-1	International borders	Resistance	1000	-0.367	NA
NIE-JIS-1	International borders	Resistance	10000	-0.369	NA
NIE-JIS-1	International borders	Resistance	100000	-0.369	NA
NIE-JIS-1	International borders	Conductance	10	0.031	0.01
NIE-JIS-1	International borders	Conductance	100	-0.034	NA
NIE-JIS-1	International borders	Conductance	1000	-0.201	NA

NIE-JIS-1	International borders	Conductance	10000	-0.251	NA
NIE-JIS-1	International borders	Conductance	100000	-0.257	NA

Table S3.
Metadata associated to the 38 newly generated WPV1 VP1 sequences used in this study.

ID	Lab	Source	Country of Collection	Year of Collection
12W1	MHRA	AFP	Turkey	1989
18W2	MHRA	AFP	Spain	1982
19W2	MHRA	AFP	Spain	1982
19W1	MHRA	AFP	Senegal	1986
1W1	MHRA	AFP	Mexico	1980
20W1	MHRA	AFP	Turkey	1985
20W2	MHRA	AFP	Spain	1983
21W1	MHRA	AFP	Kuwait	1980
22W1	MHRA	AFP	Kuwait	1982
23W1	MHRA	AFP	Gambia	1986
24W1	MHRA	AFP	Gambia	1986
25W1	MHRA	AFP	Greece	1976
26W1	MHRA	AFP	New York, USA	1977
2W1	MHRA	AFP	Romania	1981
31W1	MHRA	AFP	Mississippi, USA	1958
32W1	MHRA	AFP	New Mexico, USA	1957
33W1	MHRA	AFP	Alaska, USA	1959
34W1	MHRA	AFP	California, USA	1953
3W1	MHRA	AFP	Oman	1988
44W1	MHRA	AFP	Nigeria	1985
45W1	MHRA	AFP	Oman	1988
48W1	MHRA	AFP	SouthAfrica	1983
49W1	MHRA	AFP	Ghana	2003
4W1	MHRA	AFP	HongKong	1981
50W1	MHRA	AFP	Ghana	2003
51W1	MHRA	AFP	Ghana	1998
53W1	MHRA	AFP	Texas, USA	1970
58W1	MHRA	AFP	Pakistan	1990
59W1	MHRA	AFP	Singapore	1986
5W1	MHRA	AFP	Pakistan	1992
9W1	MHRA	AFP	Zaire	1983
M2	MHRA	AFP	Morocco	1977
M23	MHRA	AFP	Morocco	1978
M24	MHRA	AFP	Morocco	1979
M26	MHRA	AFP	Morocco	1978
M28	MHRA	AFP	Morocco	1979
M30	MHRA	AFP	Morocco	1978
M35	MHRA	AFP	Morocco	1979

Table S4.

Missing metadata for all poliovirus GenBank entries downloaded on 22nd July 2022 discretised by serotype.

Field	Polio 1	%	Polio 2	%	Polio 3	%	Total	%
**Isolate**	895	23.8	598	24.9	436	40.8	1929	26.7
**Host**	2150	57.2	700	29.2	479	44.8	3329	46.1
**Country**	930	24.7	153	6.4	139	13.0	1222	16.9
**Subnational**	3488	92.8	2238	93.3	955	89.3	6681	92.4
**Source**	2758	73.4	1580	65.9	591	55.3	4929	68.2
**Collection date**	1825	48.5	563	23.5	282	26.4	2670	36.9
**Strain**	2510	66.8	1255	52.3	578	54.1	4343	60.1
**Serotype**	2828	75.2	1048	43.7	598	55.9	4474	61.9
**Total entries (N)**	3760		2398		1069		7227	

Table S5. Information on clock models used for each dataset in the study.

Dataset	UCLD rate stats		Final clock model
	Standard deviation	Coefficient of variation	
A - Global	7.6x10-3 (6.5-8.8x10-3)	0.66 (0.6-0.72)	UCLD
B - Clade 3	4.1x10-3 (2.7-5.6 x10-3)	0.391 (0.28-0.51)	UCLD
C - Clade 4a	1.2x10-4 (4.9x10-11-3.55x10-4)	2.0x10-2 (4.09x10-15-0.06)	Strict
D - Clade 4b	8.1x10-4 (1.9x10-4-1.41x10-3)	0.1 (0.02-0.17)	Strict
E - WPV3	8.8x10-5 (2.1x10-8-2.5x10-4)	1.2x10-2 (3.3x10-6-0.03)	Strict

Table S6. Additional information on the specifications used for phylogenetic and phylogeographic analysis in BEAST for each dataset.

Dataset	Size	Skygrid grids	Frequency of grids	Last transition	Chain Length ($x10^6$)	Replicates	Chain Length (DTA)($x10^6$)	Replicates DTA
A - Global	1572	75	1/year	75	300	3	20	2
B - Clade 3	201	74	2/year	37	50	2	50	2
C - Clade 4a	304	20	1/year	20	100	2	10	2
D - Clade 4b	559	16	1/year	12	100	2	10	2
E - WPV3	123	50	1/year	50	20	2	30	2
