# Supplementary material for: Historical and current spatiotemporal patterns of wild and vaccine-derived poliovirus spread
Source: Nat Microbiol. 2025 Nov 27;10(12):3148–61. doi: 10.1038/s41564-025-02174-6 (PMC12669034; doi:10.1038/s41564-025-02174-6)
Supplement: Supplementary file 2 — Reporting Summary [file 41564_2025_2174_MOESM2_ESM.pdf]

nature portfolio

Corresponding author(s): Darlan Da Silva Candido

Last updated by author(s): Sep 26, 2025

Reporting Summary

Nature Portfolio wishes to improve the reproducibility of the work that we publish. This form provides structure for consistency and transparency in reporting. For further information on Nature Portfolio policies, see our Editorial Policies and the Editorial Policy Checklist.

Statistics

For all statistical analyses, confirm that the following items are present in the figure legend, table legend, main text, or Methods section.

n/a	Confirmed	
☐	☒	The exact sample size (*n*) for each experimental group/condition, given as a discrete number and unit of measurement
☒	☐	A statement on whether measurements were taken from distinct samples or whether the same sample was measured repeatedly
☐	☒	The statistical test(s) used AND whether they are one- or two-sided<br>*Only common tests should be described solely by name; describe more complex techniques in the Methods section.*
☐	☒	A description of all covariates tested
☐	☒	A description of any assumptions or corrections, such as tests of normality and adjustment for multiple comparisons
☐	☒	A full description of the statistical parameters including central tendency (e.g. means) or other basic estimates (e.g. regression coefficient) AND variation (e.g. standard deviation) or associated estimates of uncertainty (e.g. confidence intervals)
☒	☐	For null hypothesis testing, the test statistic (e.g. *F*, *t*, *r*) with confidence intervals, effect sizes, degrees of freedom and *P* value noted<br>*Give P values as exact values whenever suitable.*
☐	☒	For Bayesian analysis, information on the choice of priors and Markov chain Monte Carlo settings
☒	☐	For hierarchical and complex designs, identification of the appropriate level for tests and full reporting of outcomes
☒	☐	Estimates of effect sizes (e.g. Cohen's *d*, Pearson's *r*), indicating how they were calculated

Our web collection on statistics for biologists contains articles on many of the points above.

Software and code

Policy information about availability of computer code

Data collection	Epidemiological data was downloaded from POLIS.
Data analysis	Data visualization was performed using ggplot2 in R. Wavefront velocity analysis was performed using a custom made code developed by Simon Dellicour based on the use of circuitscape 5 in R (the code has been made available on github: https://github.com/sdellicour/vdpv_wavefront). For sequencing and phylogenetic analysis, all softwares used are available for free: Guppy v5, Geneious 10.2.3, BEAST v.1.10.5, BEAGLE v4.0.0 , IQTREE v.2 , FastTree v.2.1, Tracer v1.7.2, FigTree v1.4.4, TempEst v.1.5.3, TreeAnnotator 1.10.4 , LogCombiner 1.10.4 60, and AliView 1.28.

For manuscripts utilizing custom algorithms or software that are central to the research but not yet described in published literature, software must be made available to editors and reviewers. We strongly encourage code deposition in a community repository (e.g. GitHub). See the Nature Portfolio guidelines for submitting code & software for further information.

Data

Policy information about availability of data

All manuscripts must include a data availability statement. This statement should provide the following information, where applicable:

- Accession codes, unique identifiers, or web links for publicly available datasets
- A description of any restrictions on data availability
- For clinical datasets or third party data, please ensure that the statement adheres to our policy

Genetic sequences are available on GenBank under the following accession numbers: PQ159203-PQ159240. Detailed disease surveillance data on which this research is based are available from the WHO Institutional Data Access/Ethics Committee for Global Polio Eradication Initiative research partners who meet the criteria for access to confidential data.

Research involving human participants, their data, or biological material

Policy information about studies with human participants or human data. See also policy information about sex, gender (identity/presentation), and sexual orientation and race, ethnicity and racism.

Reporting on sex and gender	Our study focuses on the spatiotemporal patterns of poliovirus spread rather than on demographic and clinical characteristics of infected patients and only uses surveillance data from the WHO POLIS.Epidemiological data on all acute flaccid paralysis(AFP) cases with paralysis onset after "The Switch", May 1, 2016, through September 29, 2023, was downloaded from the Polio Information System (POLIS). Sequences were obtained from historical WPV1 isolates available at Medicines and Healthcare products Regulatory Agency (MHRA), including viruses from 1953 to 2011 from all WHO regions and some which had been stored at MHRA for more than 40 years and demographic information is not available.
Reporting on race, ethnicity, or other socially relevant groupings	Please see above.
Population characteristics	Please see above.
Recruitment	No recruitment was performed as this is a study based on surveillance data. Epidemiological data on all acute flaccid paralysis(AFP) cases with paralysis onset after "The Switch", May 1, 2016, through September 29, 2023, was downloaded from the Polio Information System (POLIS). For the epidemiological data, potential biases are related to differences in the surveillance capacity and reporting across different countries. Sequences were obtained from historical WPV1 isolates available at Medicines and Healthcare products Regulatory Agency (MHRA), including viruses from 1953 to 2011 from all WHO regions and some which had been stored at MHRA for more than 40 years.
Ethics oversight	Imperial College Research Governance and Integrity Team (reference ID 21IC6996)

Note that full information on the approval of the study protocol must also be provided in the manuscript.

Field-specific reporting

Please select the one below that is the best fit for your research. If you are not sure, read the appropriate sections before making your selection.

☒ Life sciences ☐ Behavioural & social sciences ☐ Ecological, evolutionary & environmental sciences

For a reference copy of the document with all sections, see nature.com/documents/nr-reporting-summary-flat.pdf

Life sciences study design

All studies must disclose on these points even when the disclosure is negative.

Sample size	Study based on surveillance data. All available surveillance data was used for analysis and, as such, no sample size calculation was performed. Epidemiological data on all acute flaccid paralysis (AFP) cases with paralysis onset after "The Switch", May 1, 2016, through September 29, 2023, were downloaded from the Polio Information System (POLIS),. Briefly, the polio surveillance system involves the surveillance of AFP cases, environmental samples, contact sampling and community sampling. Poliomyelitis cases are laboratory confirmed according to WHO guidelines using virus isolation in cell culture, intertypic differentiation and genetic sequencing of the VP1 capsid protein.
Data exclusions	No exclusions were performed. All available epidemiological and genomic data was used meeting the minimum criteria specified in methods was used. For genomic data, only sequences including the VP1 region of WPV1 poliovirus, used routinely for surveillance, and for which data on country and collection data were available were used. Please see methods.
Replication	Study based on surveillance data. Not applicable.
Randomization	Study based on surveillance data. Not applicable.
Blinding	Study based on surveillance data. Not applicable.

Reporting for specific materials, systems and methods

We require information from authors about some types of materials, experimental systems and methods used in many studies. Here, indicate whether each material, system or method listed is relevant to your study. If you are not sure if a list item applies to your research, read the appropriate section before selecting a response.

Materials & experimental systems

n/a	Involved in the study
☒	☐ Antibodies
☒	☐ Eukaryotic cell lines
☒	☐ Palaeontology and archaeology
☒	☐ Animals and other organisms
☒	☐ Clinical data
☒	☐ Dual use research of concern
☒	☐ Plants

Methods

n/a	Involved in the study
☒	☐ ChIP-seq
☒	☐ Flow cytometry
☒	☐ MRI-based neuroimaging

Plants

Seed stocks	*Report on the source of all seed stocks or other plant material used. If applicable, state the seed stock centre and catalogue number. If plant specimens were collected from the field, describe the collection location, date and sampling procedures.*
Novel plant genotypes	*Describe the methods by which all novel plant genotypes were produced. This includes those generated by transgenic approaches, gene editing, chemical/radiation-based mutagenesis and hybridization. For transgenic lines, describe the transformation method, the number of independent lines analyzed and the generation upon which experiments were performed. For gene-edited lines, describe the editor used, the endogenous sequence targeted for editing, the targeting guide RNA sequence (if applicable) and how the editor was applied.*
Authentication	*Describe any authentication procedures for each seed stock used or novel genotype generated. Describe any experiments used to assess the effect of a mutation and, where applicable, how potential secondary effects (e.g. second site T-DNA insertions, mosiacism, off-target gene editing) were examined.*
